# A Study on the Material Removal Characteristics and Damage Mechanism of Lapping for Pressureless Sintered Silicon Carbide (SSiC) Microlens Cavity

**DOI:** 10.3390/mi14061162

**Published:** 2023-05-31

**Authors:** Tianfeng Zhou, Zhongyi Li, Weijia Guo, Peng Liu, Bin Zhao, Xibin Wang

**Affiliations:** 1 School of Mechanical Engineering, Beijing Institute of Technology, No. 5 Zhongguancun South Street, Haidian District, Beijing 300081, China; zhoutf@bit.edu.cn (T.Z.); 3120200348@bit.edu.cn (Z.L.); lpamt@bit.edu.cn (P.L.); bin.zhao@bit.edu.cn (B.Z.); cutting0@bit.edu.cn (X.W.); 2 Beijing Institute of Technology Chongqing Innovation Center, Chongqing 401120, China

**Keywords:** microlens, lapping, precision machining, material removal mechanism

## Abstract

Microlens arrays have been widely employed to control the reflection, refraction, and diffraction characteristics of light due to its distinctive surface properties. Precision glass molding (PGM) is the primary method for the mass production of microlens arrays, of which pressureless sintered silicon carbide (SSiC) is a typical mold material due to its excellent wear resistance, high thermal conductivity, high-temperature resistance, and low thermal expansion. However, the high hardness of SSiC makes it hard to be machined, especially for optical mold material that requires good surface quality. The lapping efficiency of SSiC molds is quite low. and the underlying mechanism remains insufficiently explored. In this study, an experimental study has been performed on SSiC. A spherical lapping tool and diamond abrasive slurry have been utilized and various parameters have been carried out to achieve fast material removal. The material removal characteristics and damage mechanism have been illustrated in detail. The findings reveal that the material removal mechanism involves a combination of ploughing, shearing, micro-cutting, and micro-fracturing, which aligns well with the results obtained from finite element method (FEM) simulations. This study serves as preliminary reference for the optimization of the precision machining of SSiC PGM molds with high efficiency and good surface quality.

## 1. Introduction

Pressureless sintered silicon carbide (SSiC) is a typical mold material for precision glass molding (PGM) due to its good wear resistance, high thermal conductivity, high-temperature resistance, and low thermal expansion [1,2]. It is fabricated using submicron β-SiC powder with a small amount of B and C addition at a high temperature between 1900 °C and 2200 °C, making it possible to use SSiC across the entire pH-value [3]. Despite these advantages, it is difficult to seek a suitable manufacturing technology and process to machine such hard-to-machine materials, especially for those machining methods with high precision and high efficiency. The underlying material removal characteristics and damage mechanism have not been fully understood. Commonly used machining processes for SSiC include grinding, lapping, and polishing [4]. Studies have been conducted on SiC ceramics machining to study the underlying mechanism during the machining process. A SiC ceramics model was established by Gu et al. and they revealed through experiments that the surface roughness could be reduced to 152 nm with an abrasive particle size of 0.5 μm and polishing depth of 2 μm [5]. Liu et al. performed the polishing on SSiC ceramics and found that the ductile material removal mechanism could be achieved using the abrasive particle size of 1 μm [6]. Dong et al. investigated the fixed abrasive diamond lapping process of SiC and found that the subsurface damage layer highly depends on the diamond particle size [7]. Li et al. studied the surface quality and subsurface damage evolution on RB-SiC [8]. Although it has been proven that a high material rate can be obtained through lapping, all of the above-mentioned studies lay a good foundation for the further research. However, most of the previous studies have primarily focused on flat surface material removal. While lapping and polishing techniques are commonly associated with achieving smooth and flat surfaces, there have been notable advancements in generating structured surfaces using these methods. For efficient fabrication of microlens arrays on hard mold surfaces, Liu et al. employed precision balls and diamond slurries in their study. They successfully fabricated microlens arrays on both concave and convex surfaces using a novel stage-based lapping process facilitated by a self-developed lapping system on a silica surface [9]. Zhang et al. proposed a two-step manufacturing method for microlens arrays that combined microindentation and precision polishing. This approach proved effective for fabricating spherical microlens array molds [10]. Huang et al. explored the generation of multiscale dimples on metallic glass through polishing and subsequent nanoindentation, which demonstrated the feasibility of using polishing techniques in combination with nanoindentation to create structured surfaces [11]. While the aforementioned studies highlight the expanding capabilities of lapping and polishing techniques in achieving structured surfaces and fabricating microlens arrays, there remains a research gap in understanding the material removal characteristics and damage mechanisms specific to the lapping process of a microlens, especially on pressureless sintered silicon carbide (SSiC).

To gain insights into the interaction between the lapping tool, abrasive slurry, and microlens mold surface, the present study involves experiments on microlens cavity lapping conducted on SSiC mold material using diamond abrasive slurry with different particle sizes to explore the surface morphology evolution, material removal characteristics, and damage mechanism. The surface morphology and surface topography have been observed and characterized using scanning electron microscopy (SEM) and 3D laser confocal scanning microscopy. A model of the surface interaction between the lapping tool and SSiC workpiece has been simulated using AdvantEdge FEM. The material removal characteristics and damage mechanism have been illustrated in detail. This study can provide preliminary reference for the precision machining of SSiC PGM molds both experimentally and theoretically.

## 2. Experimental Details

**Figure 1:**
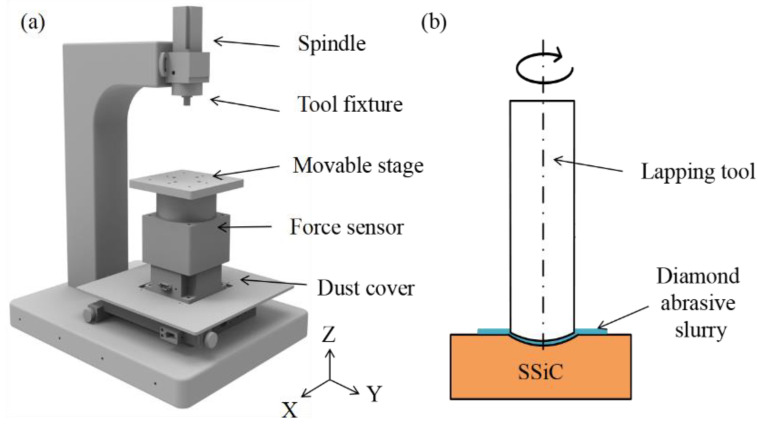
Schematic diagram of the (**a**) self-developed lapping machine and (**b**) lapping process of a SSiC microlens cavity using diamond slurry.

The experiments were performed on a self-developed lapping platform, as shown in Figure 1. As shown in Figure 1a, the overall structure of the platform is C-shaped with a marble stage to ensure the rigidity of the platform. Grating rulers are installed on platforms X, Y, and Z for precise control in three directions and to ensure movement along the X, Y, and Z axes. A force sensor has been installed to measure the lapping pressure during the machining process. The servo motor relates to the spindle. The lapping tool is fixed to the chuck tool fixture. Thus, the rotation movement of the lapping tool is driven by the spindle. At the same time, diamond abrasive slurry is sprayed on the workpiece regularly during the microlens cavity lapping process. 

**Figure 2:**
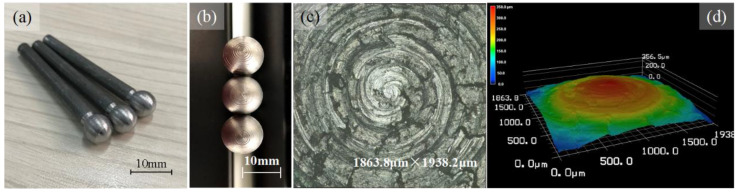
(**a**) Photograph of the lapping tool. (**b**) Photograph of the lapping tool head. (**c**) Magnificent surface morphology of the lapping tool head. (**d**) Three-dimensional surface morphology of the lapping tool head.

**Figure 3:**
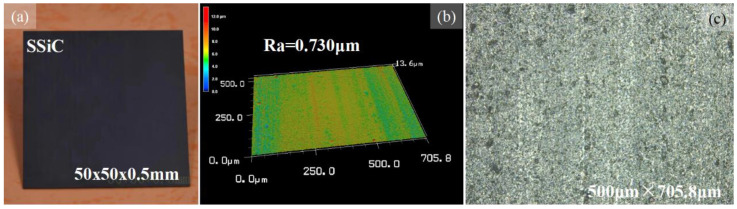
(**a**) Photograph of the SSiC workpiece. (**b**) Three-dimensional surface morphology of the SSiC workpiece. (**c**) Magnificent surface morphology of the SSiC workpiece.

A cemented tungsten carbide (WC) spherical lapping tool head has been used in this study, as shown in Figure 2. The photograph of the lapping tool and enlarged lapping tool head are shown in Figure 2a,b. The diameter of the lapping tool head is 10 mm. The surface morphology and 3D topography are depicted in Figure 2c,d. The lapping tool surface is very rough with bumps and hollows covering the surface. The intentional design of the lapping tool head is for storing the abrasive slurry in order to facilitate the material removal and extract the chips. Meanwhile, this structure can help to release heat. During the lapping process, the movement of the cemented tungsten carbide lapping tool will drive the abrasive particles and realize the material removal, while the shapes of the cavities are replicated from the tool head. The material used in this study is pressureless sintered silicon carbide (SSiC) with a size of 15 mm × 15 mm, a thickness is 0.5 mm cut from a large piece of 50 mm × 50 mm, as shown in Figure 3 and supplied by Fuzhou Pengkun Optoelectronic Technology Co., Ltd. (Fuzhou, China). The SSiC is brittle and hard, its hardness is measured to be greater than Hs 115, and its elastic modulus is 410 GPa. The surface roughness is Ra~0.730 μm. 

During the experiments, the SSiC workpiece is fixed onto the stage. The experimental parameters are listed in Table 1. The rotational speed of the lapping tool is 720 degree/s. The feed rate of the drilling is 0.5 μm/s. The material removal in thickness is set at 30 μm. The composition of the diamond abrasive slurry, as provided by the supplier, is abrasive (6%), thickening agent (20%), dispersant (24%), lubricant (16%), blending agent (22%), and lapping assisting agent (12%). The applied specifications of the abrasive slurry are W7 and W20, respectively, of which the particle sizes are 5.0–6.0 μm and 15.0–17.0 μm, accordingly. For the self-developed lapping machine, a contact pressure of 0.1 N is set as the starting point. The lapping process begins when the lapping tool contacts the workpiece at the starting point. When the lapping tool contacts the workpiece, the contact pressure is relatively low. The pressure gradually increases as the lapping process progresses to achieve the desired depth of the material removal. The pressure can be monitored during the lapping process. As the lapping tool is driven down, the pressure increases to maintain a consistent material removal rate to achieve the desired depth. The lapping time is the sum of the desired depth/feed rate and bottom delay time.

## 3. Results and Discussions

### 3.1. Finite Element (FE) Modeling of the Microlens Cavity Lapping Process

It is known that two-body or three-body wear are two typical contact wear mechanisms [12]. In the lapping process, the abrasive particle is pressed by both the WC lapping tool and the SSiC surface and may be embedded inside the lapping tool, leading to scratches and grooves on the SSiC surface, similar to the micro grinding process [13]. However, with a longer machining duration, the two-body wear mode is weakened by the wear of the abrasive particle itself and it gradually transfers to the three-body wear mode, of which the small abrasive particles impact the surface, leading to surface cracks, fracture pits, and discrete breakouts [14]. In this study, the utilization of a WC lapping tool head results in a larger exposed diamond abrasive particle surface and allows for a more aggressive material removal and faster lapping, compared to using a softer lapping tool. Therefore, the material removal is more effective.

The self-defined constitutive model is employed to characterize the behavior of the material, thereby encompassing the determination of the mold properties. The material’s yield and failure criteria are established using the Drucker–Prager criterion that considers various factors such as hydrostatic pressure, work hardening, strain rate, and thermal softening [15]. This criterion is expressed as:

(1) $$\sigma (\varepsilon^{\prime \prime },\dot{\varepsilon },T)=g\left(\varepsilon^{\prime \prime }\right)\times \Gamma \left(\dot{\varepsilon }\right)\times \theta \left(T\right)$$

where $g\left(\varepsilon^{\prime \prime }\right)$ is the working hardening function, $\Gamma \left(\dot{\varepsilon }\right)$ is the strain rate function, and $\theta \left(T\right)$ is the thermal softening function.

When $\varepsilon_{cut}^{p}\leq \varepsilon^{p}$, the working hardening function can be defined as:

(2) $$g\left(\varepsilon^{\prime \prime }\right)=\sigma_{0}{\left(1+\frac{\varepsilon_{cut}^{p}}{\varepsilon^{p}}\right)}^{\frac{1}{n}}$$

Among which, $\sigma_{0}$ is the initial yield stress, $\varepsilon^{p}$ is the plastic strain, $\varepsilon_{0}^{p}$ is the reference plastic strain, $\varepsilon_{cut}^{p}$ is the strain value at fracture, and n is the strain hardening coefficient.

When $T<T_{cut}$, the thermal softening function is defined as:

(3) $$\theta \left(T\right)=c_{0}+c_{1}T+c_{2}T^{2}+c_{3}T^{3}+c_{4}T^{4}+c_{5}T^{5}$$

When $T\geq T_{cut}$, the thermal softening function is defined as:

(4) $$\theta \left(T\right)=\theta \left(T_{\mathrm{cut}\;}\right)\left(1-\frac{T-T_{\mathrm{cut}\;}}{T_{\mathrm{melt}\;}-T_{\mathrm{cut}\;}}\right)$$

Since the rotational speed in the lapping process is low and the abrasive particles are randomly distributed on the workpiece, the influence of temperature on the processing is not considered.

When $\dot{\varepsilon }\leq \dot{\varepsilon_{t}}$, the strain rate is defined as:

(5) $$\Gamma \left(\dot{\varepsilon }\right)={\left(1+\frac{\dot{\varepsilon }}{\dot{\varepsilon_{0}}}\right)}^{\frac{1}{m_{1\;}}}$$

While $\dot{\varepsilon }>\dot{\varepsilon_{t}}$, the strain rate is defined as:

(6) $$\Gamma \left(\dot{\varepsilon }\right)={\left(1+\frac{\dot{\varepsilon }}{\dot{\varepsilon_{0}}}\right)}^{\frac{1}{m_{2}}}{\left(1+\frac{\dot{\varepsilon_{t}}}{\dot{\varepsilon_{0}}}\right)}^{\left(\frac{1-1}{m_{1\;}-m_{2\;}}\right)}$$

where $\dot{\varepsilon }$ is the strain rate, $\dot{\varepsilon_{0}}$ is the reference plastic strain rate, $\dot{\varepsilon_{t}}$ is the critical strain rate for the transition between high and low strain rates, $m_{1}$ is the low strain rate sensitivity coefficient, and $m_{2}$ is the high strain rate sensitivity coefficient.

**Figure 4:**
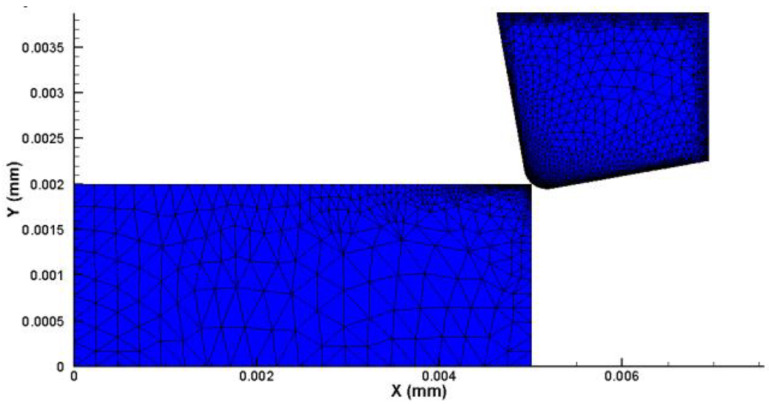
The initial grid of the abrasive tool and workpiece.

**Figure 5:**
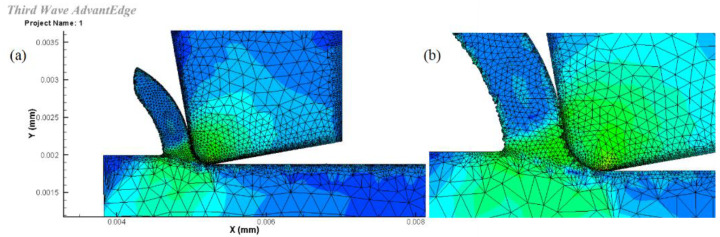
(**a**) Abrasive particles, SSiC wafers, and chips during the single abrasive micro-cutting process. (**b**) Enlarged area of the contact point.

Based on the above theory, the interactions between the spherical WC lapping tool diamond abrasive slurry and the workpiece were conducted. An FE model of a single abrasive diamond cutting process was developed for this study using Advantedge FEM. It is assumed that the abrasive particle is a rigid body because of its high hardness. The abrasive wear is ignored. The unit is defined as a solid six node equilateral triangle that is meshed using continuous adaptive mesh generation. The tool is also represented by a rigid body and its behavior is described using linear elastic law. The initial grid of the tool and workpiece is shown in Figure 4. A fine and dense grid is set on the surface of the workpiece, with a minimum grid size of 5 nm in order to analyze the processed surface. The friction between the abrasive particles and the workpiece is set to comply with Coulomb’s friction law. The simulation model of the single abrasive micro-cutting process is illustrated in Figure 5. 

The analysis reveals the presence of distinct deformation zones and the occurrence of continuous chip formation during the machining process. Notably, as the abrasive particles plow into the SSiC surface, plastic deformation occurs, causing the material to flow and undergo plastic deformation. This plastic deformation plays a crucial role in smoothing the surface and refining the surface finish. Based on the single abrasive diamond micro-cutting finite element (FE) model, it becomes evident that the material removal of the abrasive grain embedded in the workpiece surface is primarily governed by plastic rheology. The material undergoes plastic deformation and plastic flow, resulting in the removal and accumulation of material accompanied by the formation of bumps and ploughing trails. Comparatively, plastic working offers advantages over brittle fracture as it contributes to the attainment of a smoother surface. The findings highlight the importance of understanding the material removal mechanism during the lapping process using multiple abrasives to obtain a smoother surface finish and enhance the overall quality of the SSiC microlens molds.

### 3.2. Surface Morphology and Elemental Composition Characterization

**Figure 6:**
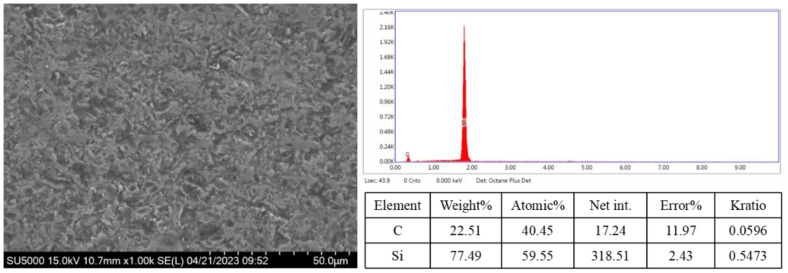
SEM surface morphology and EDX elemental composition.

SEM (Hitachi SU5000, Hitachi, Ltd., Tokyo, Japan) was used to observe the microscopic surface morphology of the SSiC workpiece. The SEM image of the pristine SSiC is shown in Figure 6. Although the surface is very rough, no large pores or cracks can be observed. Energy dispersive X-ray spectroscopy (EDX) was performed to measure the surface elemental composition. The surface is mainly composed of carbon (C) (40.54%, atomic percentage) and silicon (Si) (59.55%, atomic percentage).

**Figure 7:**
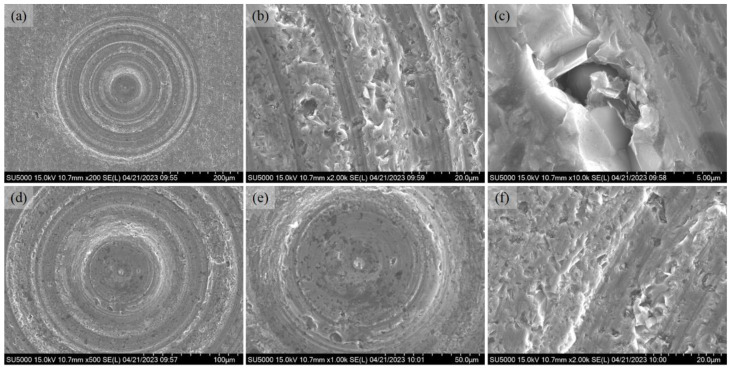
SEM images of the lapping microlens cavity surface (abrasive slurry: W20, bottom delay: 100 s, first round). (**a**) Entire cavity. (**b**) Enlarged lapping trails. (**c**) Enlarged breakout area. (**d**) Enlarged image. (**e**) Enlarged center area. (**f**) Enlarged lapping trails.

**Figure 8:**
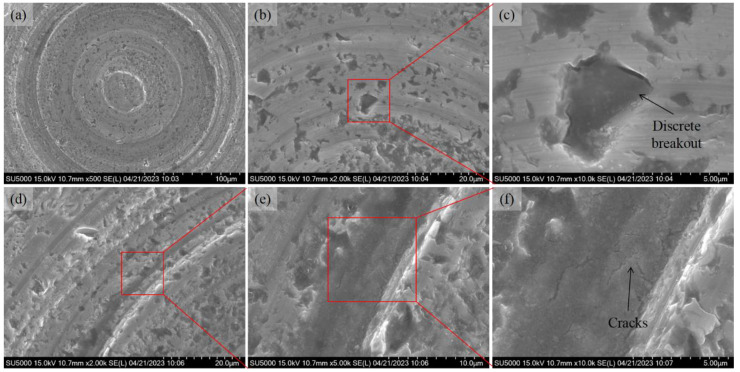
SEM images of the lapping microlens cavity surface (abrasive slurry: W20, bottom delay: 100 s, second round). (**a**) Entire cavity. (**b**) Enlarged lapping trails. (**c**) Enlarged breakout area. (**d**) Enlarged local area. (**e**) Enlarged trails. (**f**) Enlarged cracks.

**Figure 9:**
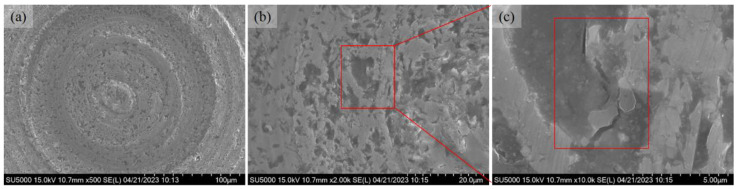
SEM images of the lapping microlens cavity surface (abrasive slurry: W20, bottom delay: 100 s, third round). (**a**) Entire cavity. (**b**) Enlarged lapping trails. (**c**) Enlarged surface cracks.

Three microlens cavities were machined consecutively using the same lapping tool without any replacement parts. The experimental parameters remained consistent throughout the process including the usage of W20 abrasive slurry and a bottom delay of 100 s. The surface morphology of each cavity is presented in Figure 7, Figure 8 and Figure 9, respectively. These figures show the resulting surface characteristics achieved. 

Figure 7 shows the surface morphology of the lapping surface (abrasive slurry: W20, bottom delay: 100 s). It clearly shows that the microlens cavity is full of lapping trails. When the lapping tool is swept on the workpiece surface, the diamond abrasive particle applies a lapping force on SSiC, and as the tool moves across the SSiC surface, it ploughs and shears the surface, causing material to be removed in small chips. As shown in Figure 7b,f, small pits appear after lapping and the local pits can become enlarged, as in Figure 7c. This might be due to the micropores formed during the sintering process. Another possible reason is due to the material removal of small chips. In addition, the lapping trails are shown with different brightness and are not uniform with different diameters. When lapping SSiC with diamond abrasive particles, the hard diamond abrasive particles create a cutting action against the SSiC surface and remove the material through a combination of micro-cutting and micro-fracturing mechanisms. The diamond abrasive particles can also generate new sharp cutting edges through fracturing during the lapping process, which helps to maintain a consistent material removal rate. It is also noteworthy that at the center location of the cavity, as shown in Figure 7e, there is little material removal in this area. The reason for this phenomenon is that the relative local speed is zero, meaning that the diamond abrasive cannot take part in the lapping machining.

As shown in Figure 8, one more microlens cavity has been machined using the same experimental parameters, without any tool replacements. The surface morphology of the entire cavity and the locally enlarged area is shown. Similar to the first cavity, the surface is full of lapping trails along with micropores on the surface after lapping. It is noteworthy that more micropores appeared and a discrete breakout occurred during the lapping process, meaning that the brittle removal of SiC was dominant in the material removal of this cavity, as shown in Figure 8b and enlarged in Figure 8c. Meanwhile, there are many pits along the lapping trails. The diamond abrasive particles are firstly pressed tightly against the workpiece surface. The elastic deformation is released suddenly when the abrasive particle is in contact with a pit edge. Then, the abrasive particle continues to move forward and crush the surface. Brittle cracks are also formed, as shown enlarged in Figure 8e,f. 

Another microlens cavity has been machined using the same tool, as shown in Figure 9. The lapping trails can be observed to be much shallower, compared with Figure 7 and Figure 8. This might be due to the wear of the lapping tool head itself. As the tool becomes worn, there are some changes in the shape and size of the tool, which can affect the material removal rate and the resulting surface finish. The changes in the tool geometry can alter the distribution of pressure and the path of the tool over the SSiC surface. This can lead to uneven material removal and surface finish, as well as increased surface roughness and waviness. In addition, it may lead to the formation of burrs or defects on the SSiC surface that can negatively impact the final product quality, causing the formation of scratch marks on the SSiC surface. The brittle cracks, micropores, and pits can still be clearly seen on the lapping surface, as shown enlarged in Figure 9c.

**Figure 10:**
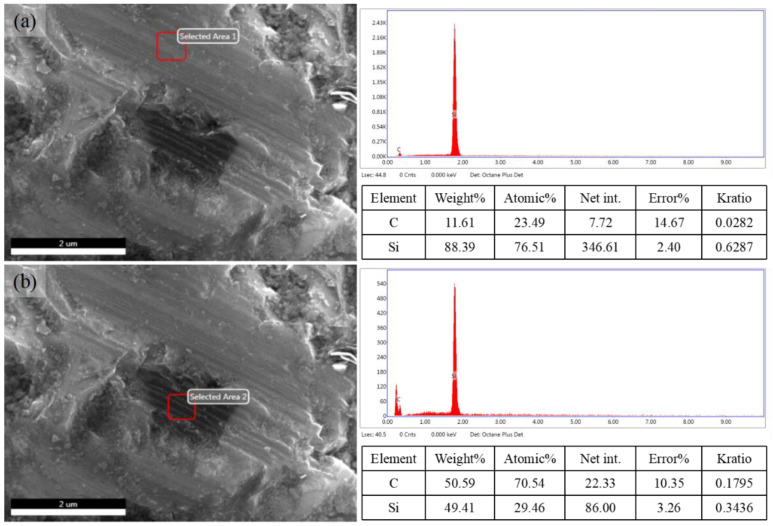
(**a**) Elemental composition of the locally enlarged lapping surface (abrasive slurry: W20, bottom delay: 100 s, third round), normal trails area. (**b**) Elemental composition of the locally enlarged lapping surface (abrasive slurry: W20, bottom delay: 100 s, third round), darker area.

It has also been observed that there are local dark areas on the lapping surface, as shown in Figure 10a,b. The EDX spectra indicate that the carbon content can be increased to 70.54% (atomic percentage) after lapping, compared to the normal trail area 23.49%. One possible reason may be due to the formation of a hardened layer on the workpiece surface. During the SSiC lapping process, the local high temperature generated by the friction between the diamond abrasive particles and SSiC can cause the carbon atoms in the material to diffuse towards the surface. This can lead to the formation of a hardened layer on the workpiece surface that may contain higher levels of carbon element than the bulk of the material. Another possible reason is that SSiC lapping can create micro-cracks and other defects in the material surface. These defects can cause carbon to be trapped and concentrated in the surface layer, leading to an apparent increase in the carbon content.

### 3.3. Surface Topography Characterization

**Figure 11:**
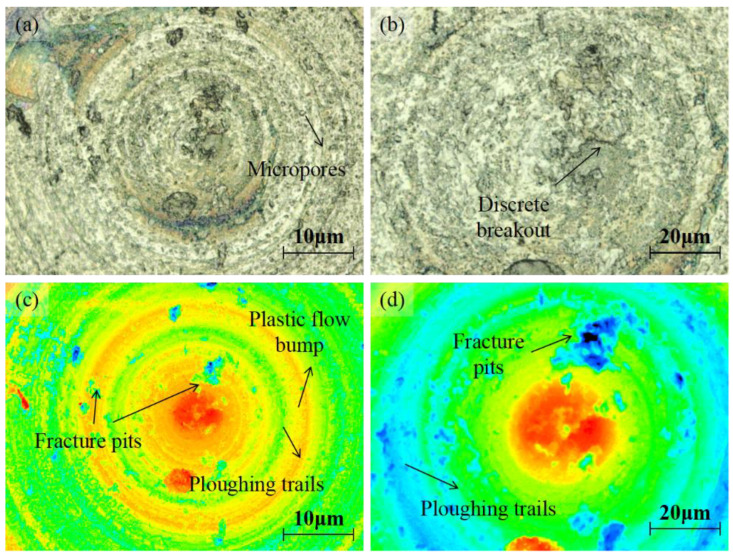
Three-dimensional laser confocal scanning microscopy image of the lapping microlens cavity surface (abrasive slurry: W7, bottom delay: 100 s). (**a**) Surface image. (**b**) Enlarged surface image. (**c**) Colored surface height image. (**d**) Enlarged image of the colored surface height image.

The surface topography of the machined microlens cavity (abrasive slurry: W7, bottom delay: 100 s) was measured using 3D laser confocal scanning microscopy, as shown in Figure 9. The results correspond well to the surface morphology change. It can be observed that micropores and discrete breakouts happened on the lapping surface. Fracture pits can be observed as well. Apart from these characteristics, plastic flow bumps and ploughing trails are shown with height variation. Since SSiC is a hard and brittle material, it is prone to cracking and fracturing under mechanical stress. During lapping, the lapping tool or diamond abrasive particles apply a great local pressure to SSiC that creates stresses that exceed the material’s fracture toughness. As a result, the material undergoes brittle fracture and small chips are removed from the surface. It was also found that the surface topography, including the size and distribution of chips, depends on the lapping parameters, as shown in Figure 11.

**Figure 12:**
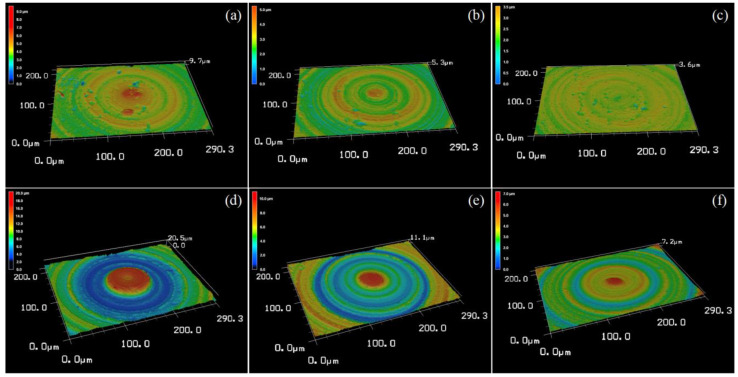
Three-dimensional laser confocal scanning microscopy image of the lapping microlens cavity surface. (**a**) Abrasive slurry: W7, bottom delay: 100 s, 1 round. (**b**) Abrasive slurry: W7, bottom delay: 100 s, 2 round. (**c**) Abrasive slurry: W7, bottom delay: 100 s, 3 round. (**d**) Abrasive slurry: W20, bottom delay: 100 s, 1 round. (**e**) Abrasive slurry: W20, bottom delay: 100 s, 2 round. (**f**) Abrasive slurry: W20, bottom delay: 100 s, 3 round.

The 3D surface topography of the microlens cavities machined using W7 and W20 abrasives is depicted in Figure 12a–f, respectively. These figures illustrate the surface characteristics of the microlens cavities under different abrasive sizes. Throughout the machining process, a constant lapping bottom delay time of 100 s was employed. Importantly, the microlens cavities were machined consecutively without replacing the lapping tool. Based on the surface topography, it is evident that the wear of the lapping tool head can significantly impact the material removal mechanism and resulting surface topography. As demonstrated, despite utilizing the same parameters for microlens cavity machining in (a–c), there is a noticeable distinction between the initially machined surfaces and the later ones. The initial surfaces exhibit non-uniform material removal and a rougher finish when compared to the subsequent surfaces. This observation holds true for microlens cavities that were machined using larger abrasive particles as well, as shown in (d–f). 

**Figure 13:**
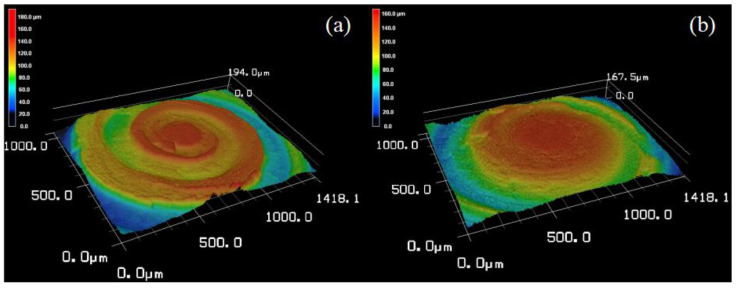
Three-dimensional laser confocal scanning microscopy image of the lapping tool surface (**a**) W7, after the machining of three cavities and (**b**) W20, after the machining of three cavities.

This phenomenon can be attributed to the surface quality of the WC lapping tool head that directly impacts the distribution of diamond abrasive particles and their interaction with the SSiC surface during the lapping process. When the lapping tool head has a rough or uneven surface, it can lead to an uneven distribution of abrasive particles. Consequently, the material removal rate becomes non-uniform, and the resulting surface finish appears rougher. Conversely, a smooth and uniform surface will allow the abrasive particles to be more evenly distributed and produce a more uniform material removal rate and a smoother surface finish. The wear of the lapping tool after machining three cavities using W7 and W20 abrasives are shown in Figure 13a,b, respectively. As the tool wears, the tool’s geometry changes and the sharpness is reduced, compared with the initial sharpness, as shown in Figure 2c. The desirable lapping process cannot be achieved either. In addition, it is found that the material removal mechanism and surface topography are affected by the abrasive particle size. The material removal volume is apparently higher when using W20 diamond abrasive slurry. The surface topography is shown to have deeper lapping trails as well. While the microlens cavity is machined using the W7 diamond abrasive size results in a lower material removal rate. In contrast, Figure 12a–c exhibit an increased presence of fracture pits, micropores, and discrete breakout features. 

These characteristics are more pronounced when smaller abrasive particles are employed during the machining process. Specifically, the smaller particles tend to create shallower and narrower scratches on the surface. Conversely, when larger abrasive particles are utilized, the resulting scratches are deeper and wider, leading to a rougher surface texture. Therefore, it can be concluded that larger diamond particles remove material mainly through a ploughing mechanism, where the particles push and displace the material. However, smaller diamond abrasive particles will remove material through a cutting mechanism, where the particles break and remove material. This corresponds to the surface topography and morphology observation.

Based on the above-mentioned results, the material removal mechanism of SSiC during the lapping process with a spherical WC lapping tool and diamond abrasive slurry is primarily brittle removal. SSiC is a hard and brittle material, meaning that it is prone to cracking and fracturing under mechanical stress. The material is removed through a combination of ploughing, shearing, micro-cutting, and micro-fracturing mechanisms, resulting from the formation and propagation of cracks and fractures in the SSiC material. During the lapping process, the spherical tungsten carbide lapping tool or diamond abrasive particles apply a high local pressure to the SSiC surface that creates stresses that exceed the material’s fracture toughness. As a result, the material undergoes brittle fracture and small chips are removed from the surface. The size and distribution of the chips depend on the properties of the SSiC material, lapping parameters, and the characteristics of the lapping tool or abrasive particles.

**Figure 14:**
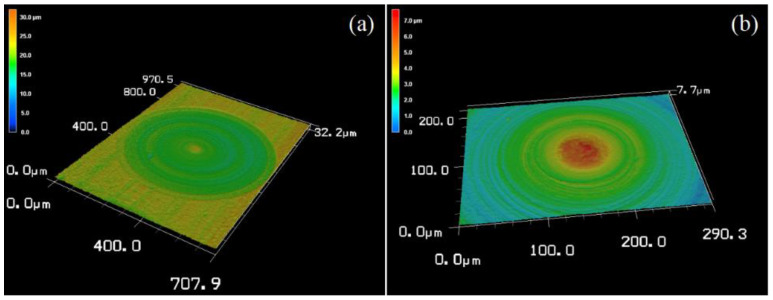
Three-dimensional laser confocal scanning microscopy image of the lapping microlens cavity surface using step lapping (abrasive slurry: W7, bottom delay: 100 s, abrasive slurry: W20, bottom delay: 100 s). (**a**) Magnification of 50. (**b**) Magnification of 100.

It was found that WC lapping tool wear and the diamond abrasive particle size can affect the material removal mechanism when lapping SSiC using a spherical WC lapping tool and diamond abrasive slurry. WC lapping tool wear can cause changes in the shape and size that can affect the material removal rate and the resulting surface finish. The size of the diamond abrasive slurry will affect the depth and texture of the scratches left on the surface. Larger abrasive particles will create deeper and wider scratches, while smaller particles will create shallower and narrower scratches. It is important to note that larger abrasive particles displace the material during lapping and create a ploughing effect. Moreover, smaller abrasive particles are less likely to cause deep scratches because they cut the material rather than displace it. Therefore, a combination of larger and smaller abrasive particles may be used to optimize both the material removal rate, material removal mechanism, and surface finish. Thus, a combination of diamond slurry with different sizes for lapping SSiC have been tried in order to improve the results. In this study, we chose W7 and W20. The microlens cavity was firstly machined using small abrasive particles (W7) that can help remove any surface defects or scratches left by the previous steps. Followed by the larger abrasive particles (W20) in order to remove the bulk material at a faster rate. The surface topography is shown in Figure 14. Despite the fact that there are a few materials around the center area, the figure clearly shows that the material removal is uniform. The micropores, discrete breakout, and fracture pits are removed. The ploughing trails are shown with high uniformity. 

However, the success of step lapping depends on various factors such as the abrasive particle size and distribution, the lapping pressure, the lapping speed, and the diamond slurry concentration. Optimization of the parameters is essential to achieve the desired surface finish and material removal rate in our future study.

## 4. Conclusions

This study focuses on the analysis of surface morphology evolution, material removal characteristics, and damage mechanism. Specifically, we revealed that the removal process involves a combination of ploughing, shearing, micro-cutting, and micro-fracturing mechanisms that align with the results obtained from finite element method (FEM) simulations. The details are as follows:

(1) WC lapping tool wear can alter the distribution of pressure and the path of the tool over the SSiC surface, and affect the material removal rate and surface quality. Lapping tool wear can lead to an uneven material removal and surface finish, as well as increase the surface roughness and waviness. In addition, it may lead to the formation of burrs or defects on the SSiC surface, causing the formation of scratch marks on SSiC surface;
(2) The diamond abrasive particle size can affect the material removal rate and surface finish. Larger diamond particles remove material mainly through a ploughing mechanism, where the particles push and displace the material. Smaller diamond abrasive particles will remove material through a cutting mechanism, where the particles break and remove material;
(3) Carbon content can be increased locally after lapping. One possible reason is the local high temperature generated by the friction between the diamond abrasive particles and SSiC, causing the carbon atoms in the material to diffuse towards the surface. Another possible reason is that SSiC lapping can create micro-cracks and defects on the surface, causing carbon to be trapped and concentrated in the surface layer; and
(4) Step lapping can improve microlens cavity lapping efficiency. Small abrasive particles can help remove any surface defects or scratches left over in the previous steps. Larger abrasive particles remove the bulk material at a faster rate.

However, there are a lot of factors that might affect the lapping process including the lapping pressure, the lapping speed, and the diamond slurry concentration. The parameters optimization will be conducted to improve the lapping efficiency and surface quality in our future study.

## Figures and Tables

**Table 1:** Experimental parameters used in this study.

Parameters	Value
Feed rate (μm/s)	0.5
Expected machining depth (μm)	30
Bottom delay duration (s)	100, 200, 300
Rotational speed (degree/s)	720
Abrasive particle size (μm)	W7, W20

## Data Availability

Not available.
